# Hard-object feeding adaptations infer relative predator-prey size relationships in Devonian placoderms

**DOI:** 10.1038/s41598-026-57261-3

**Published:** 2026-06-13

**Authors:** D. Rex Mitchell, Kate Trinajstic, John A. Long, Austin N. Fitzpatrick, Joshua Bland, Alice M. Clement

**Affiliations:** 1 College of Science and Engineering, Flinders University, Adelaide, 5042 Australia; 2 Molecular and Life Sciences, Curtin University, Bentley, Kent Street, Perth, 6102 Australia; 3 Earth and Planetary Sciences, Western Australian Museum, Perth, 6000 Australia

**Keywords:** Allometry, Arthrodire, Dirichlet normal energy, Finite element analysis, Gnathostome, Ecology, Ecology, Evolution, Zoology

## Abstract

Devonian placoderms exhibit exceptional diversity in jaw and dental morphology, making them an ideal system for examining early gnathostome feeding mechanisms. Here, we investigate inferognathal (mandible) function in eight eubrachythoracid arthrodire placoderms from Australia’s Late Devonian Gogo Formation. A combination of finite element analysis and quantification of dental surface complexity revealed distinct, size-dependent strategies for processing mechanically resistant materials (durophagy). Taxa with broad, flat dental surfaces experienced low strain during simulated bites, consistent with crushing armoured prey that could be engulfed whole. In contrast, the jaw of the largest taxon combined exceptional structural strength with highly complex, reinforced dentition, enabling force concentration to pierce through armour or shell, and tear apart prey items exceeding its oral capacity. Taxa of intermediate size exhibited higher strain and specialised slicing dentition, consistent with generalist diets or feeding strategies focused on shearing through softer tissues. Consumption of armoured prey in placoderms was therefore governed not by predator size alone, but by the relative size of prey to predator. Hard object feeding in arthrodire placoderms was not a result of a single mechanical solution, but involved several distinct solutions influenced by body size and prey-processing constraints.

## Introduction

The Late Devonian (Frasnian) Gogo Formation, located on Gooniyandi Country in the Kimberley region of northern Western Australia, provides one of the world’s most detailed windows into a complex Devonian marine ecosystem. This is because of its exceptional preservation of early arthropods, molluscs, and vertebrates^1–3^. Stem gnathostomes of the class Placodermi, an extinct group of jawed and largely armoured fishes, dominate the vertebrate fauna found at the formation^4^. Arthrodire placoderms had a distinctive jaw complex comprising paired anterior and posterior upper dental plates (supragnathals) and a pair of lower jaw bones (inferognathals) that were supported by the Meckel’s cartilage^5–7^. The inferognathals of Gogo placoderms exhibit diverse sizes, shapes, and dental configurations, suggesting a wide range of feeding functions within the ecosystem^8–10^. This diversity offers a rare opportunity to investigate feeding biomechanics and trophic differentiation during the early evolution of jawed vertebrates.

Hard object feeding (durophagy) has evolved many times in gnathostomes^11–16^, often involving high bite forces, reinforced bone structure, and specialised dental morphologies^13,17–20^. With the abundance of hard-shelled and armoured potential prey species known from Devonian waters, specialisation for hard object feeding has been proposed for several placoderm species^8,9,21^. However, even though jaw mechanics, dental complexity, and body size can play key roles in hard object feeding, their precise relationships in placoderms remain unclear.

A primary determinant of biting ability across gnathostomes is mechanical advantage , which describes the proportion of force applied by the jaw adductor muscles that is transmitted to the bite point^22^. Higher mechanical advantage, representing a capacity to generate greater bite forces from a given muscle force, is often interpreted as an adaptation for capturing larger prey or processing more mechanically resistant foods (durophagy)^14,23–26^. Previous investigations have quantified variations in mechanical advantage across placoderms, which has been argued to be influenced by both ecology and phylogeny (i.e. coccosteomorphs vs. pachyosteomorphs^5^^10^. However, mechanical advantage alone does not fully explain observed differences in jaw morphology or inferred feeding strategies.

**Fig. 1:**
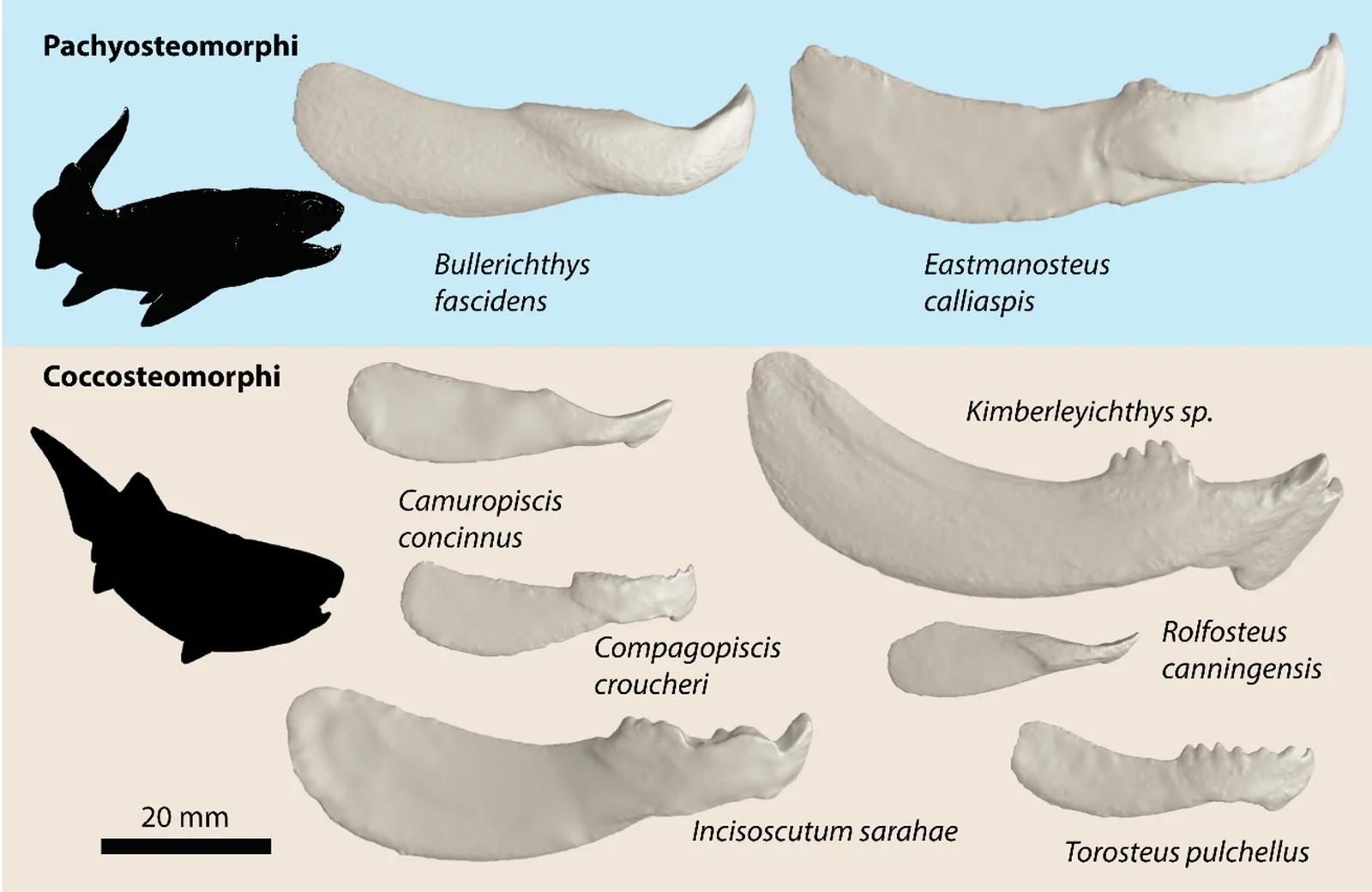
The sample of Devonian placoderm inferognathal jaw bones used in this study display ranging size and morphology reflecting their evolutionary and ecological diversity. Silhouettes not to scale.

Dental anatomy also plays a major role in predicting the diets of gnathostomes^27,28^, and is often closely tied to the mechanical demands of feeding. It was long considered that placoderms lacked “true” teeth^5^. However, the existence of true teeth with a pulp canal and dentine have since been confirmed . The evolutionary history of placoderm teeth involved the development of diverse patterns of dental organization, such as sharp slicing blades or broad, flat, edentulate surfaces^29–35^(Fig. 1). Although early studies grouped arthrodires into functional categories based on jaw shape and mechanical advantage, these groupings did not consistently align with dental morphology or inferred feeding strategies^8–10,21^. This suggests that dental morphology and bite force capacity may respond to overlapping but non-identical selective pressures. These discrepancies might be related to the variation in body size across arthrodires^3^, and its role in mediating morphological adaptation to bite force^36^.

In simplest terms, bite force is determined by the product of jaw adductor muscle force and mechanical advantage^37^. Thus, bite forces of both living and extinct species can often be estimated from skull characters, such as jaw lengths with respect to mechanical advantage^25,26,38,39^, and the presence of more robust, stiffer features to help withstand greater forces^18,19,40,41^. Dentition is also typically more robust in species that generate stronger bite forces to subdue larger prey, fracture mechanically resistant prey, or that perform biting behaviours associated with increased risk of dental damage^13,17,42^. Therefore, features such as high mechanical advantage, robust bone structure and reinforced dental arcades might be expected in hard object feeding species. However, because larger jaw muscles, more bone volume, and larger teeth (excluding filter feeders) are often intrinsic features of larger body sizes in an absolute sense, this can reduce the demands of mechanical advantage, bone reinforcement, and dental robusticity to effectively produce and withstand a given bite force^36^. For example, all else being equal, smaller species would be expected to experience greater stress and strain in the jaw than larger species when performing an identical bite force due to the less overall bone volume they have for distributing stresses^22,36^. Therefore, at least in ecological contexts, morphological adaptation to specific dietary niches is not independent of size^22^.

These considerations are particularly relevant to prey capture and processing, because bite force is so commonly used to infer predatory behaviour and prey size in gnathostomes^24,43–45^. Processing of mechanically resistant prey depends not only on bite force magnitudes, but also on how that force is applied to prey of different sizes. For example, shelled or armoured prey items that are smaller than the oral cavity can be engulfed whole, and would most likely be comminuted through distributed compression. In such cases, complex dentition is not necessary and broader, flatter dental surfaces would suffice. However, prey approaching or exceeding the predator’s oral capacity must first be mechanically fragmented into ingestible pieces. Such fragmentation might require more focused stress concentration to initiate fracture, which flatter surfaces are inherently less capable of generating. Therefore, when hard object feeding, dental complexity potentially imposed an intrinsic constraint on the maximum prey size that could be processed, especially when hard object feeding.

To test for biomechanically diverse feeding mechanisms and potential niche partitioning via prey choice in early gnathostomes, we compared inferognathal biomechanical performance and dental complexity across eight diverse species of Devonian eubrachythoracid arthrodire placoderms from the Gogo Formation. These species exhibited a suitably large range of body sizes^3^ and jaw shapes, representative of the placoderm diversity in the Gogo Formation ecosystem (Fig. 1). Because the Gogo Formation has yielded a high diversity of hard-shelled species from multiple phyla, we interpreted our findings with a focus on how jaw structure and function in arthrodires aligned with specialisation for hard object feeding.

We used finite element analysis (FEA)^22,46,47^ to simulate biting, quantify mechanical advantage, and compare resulting strain (deformation) as a proxy for jaw stiffness. Lower strain values indicated a greater capacity to withstand mechanical loading under standardised conditions^22^. Simulations involved two force standardisation techniques; one comparing equivalent bite forces relative to size (adaptive comparison), and one comparing equivalent absolute bite forces (ecological comparison)^22^. We quantified dental complexity of the inferognathal biting surface using Dirichlet Normal Energy (DNE), which measures variation in surface topography by calculating deviations in normal vectors across a mesh surface^48,49^. A normal vector is the orientation perpendicular to an element face on a mesh. A flatter surface will have more similar normal vectors, resulting in lower energy readings, while sharper or more complex topography will exhibit greater rates of change in the orientations of normal vectors, leading to higher DNE. Together, these approaches allowed us to test three hypotheses:

- H1: Inferognathal mechanical advantage is a primary determinant of bite performance.
- H2: Stiffer jaws also tend to have flatter biting surfaces, as two associated adaptations to hard-object feeding.
- H3: Including body size into models will clarify ecological distinctions.

## Results

Inferognathal lengths ranged from ~3 cm to ~7 cm, while the mechanical advantage during an anterior bite ranged approximately 6% (Table 1).

**Fig. 2:**
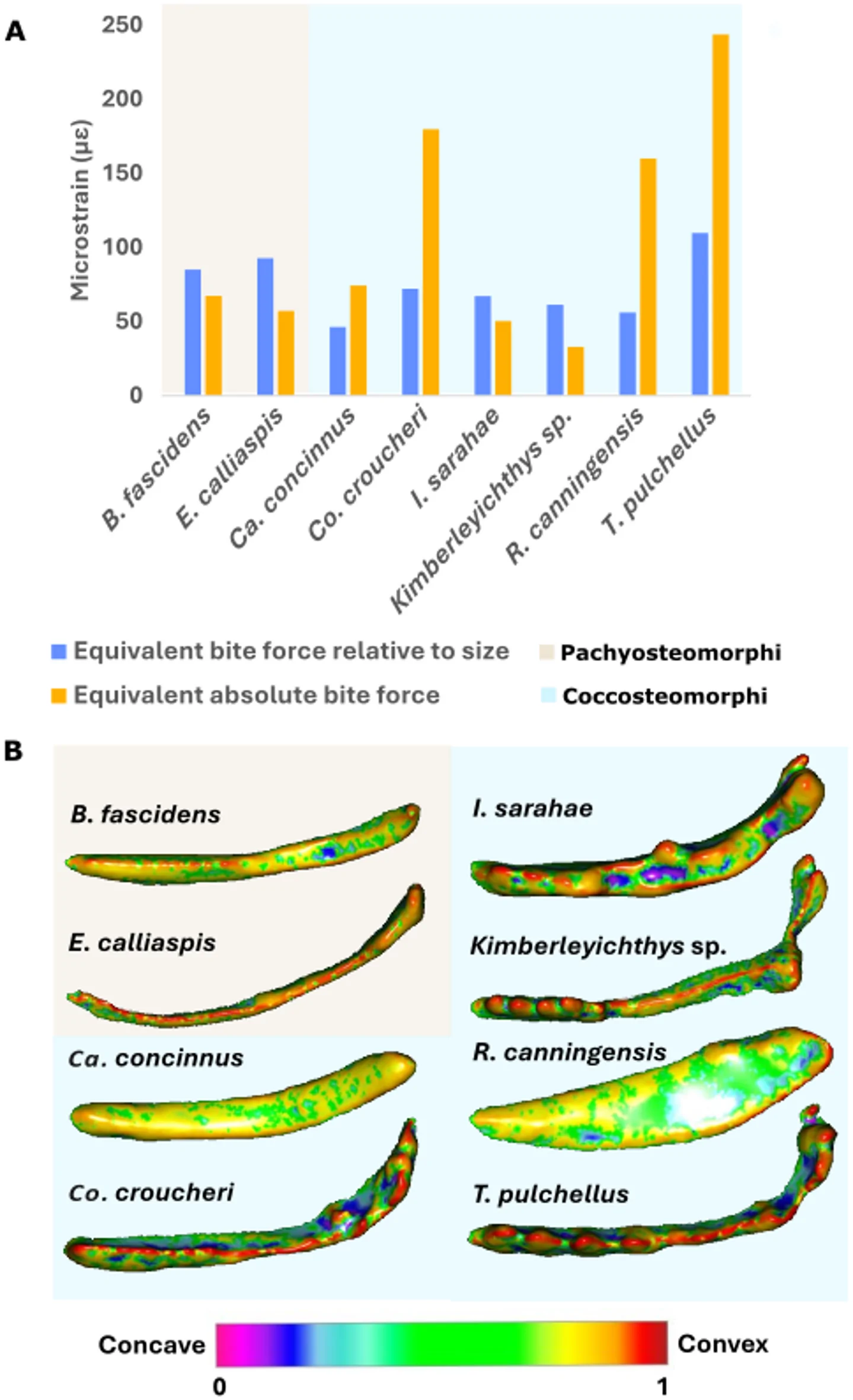
(**A**) Mean element Von Mises microstrain, experienced during a simulated bite at the front of the jaw, representing equivalent bite forces relative to size and equivalent absolute bite forces. (**B**) Dirichlet Normal Energy (DNE) surface plots for the functional dental regions of each placoderm species. Positive (red) colouration indicates sharper edges.

When first simulating equivalent bite reaction forces at the front tooth, relative to size, the lowest mean microstrain was found in *Camuropiscis concinnus* and *Rolfosteus canningensis*, and the highest in *Torosteus pulchellus* and *Eastmanosteus calliaspis* (Fig. 2A). When simulating equivalent absolute bite forces, as expected, the three species with the smallest inferognathals (see lengths in Fig. 1; Table 1), *R. canningensis*, *Compagopiscis croucheri*, and *T. pulchellus*, generated much higher mean microstrain for an identical bite force (Fig, 2 A), with *T. pulchellus* in particular attaining higher microstrain than size alone would predict. The largest species, *Kimberleyichthys* sp., had the lowest mean microstrain during equivalent bite force, also as expected. However, *Ca. concinnus* notably experienced lower microstrain than expected for its size, while *E. calliaspis* had higher microstrain than *Incisoscutum sarahae* despite being the larger of the two.

**Table 1:** Length (mm) of each inferognathal, and mechanical advantage (MA) estimated from bite simulations of each placoderm species. Dirichlet Normal Energy (DNE) was summed from concave and convex values. Length in mm.

Species	Length	MA	DNE	Convex	Concave
*B. fascidens*	55.04	0.20	145.444	138.825	6.619
*E. calliaspis*	66.03	0.23	267.044	259.476	7.568
*Ca. concinnus*	38.93	0.21	77.817	76.458	1.358
*Co. croucheri*	35.28	0.20	313.742	276.006	37.735
*I. sarahae*	62.47	0.19	242.566	209.662	32.904
*Kimberleyichthys* sp.	70.96	0.25	353.843	309.877	43.966
*R. canningensis*	29.94	0.21	116.327	111.548	4.779
*T. pulchellus*	36.05	0.25	315.953	281.868	34.085

Dirichlet Normal Energy (Table 1; Fig. 2B) was lowest in *B. fascidens*,* Ca. concinnus* and *R. canningensis*, and highest in *Kimberleyichthys* sp., *T. pulchellus*, and *Co. croucheri*. In general, flatter crushing surfaces had DNE values below ~ 150, almost entirely composed of convex curvature. Species with more complex, sharp slicing edges had values above 250, with more extreme highs of DNE relating to more complex slicing dentition, indicated by both convex and concave curvatures (Table 1; Fig. 2B). *Eastmanosteus calliaspis*, with a dental arcade consisting of a singular slicing edge, had a unique combination of both higher DNE with low concave curvature.

Inspection of both FEA simulations and DNE together revealed that lowest relative microstrain occurred alongside lowest DNE values in *Ca. concinnus* and *R. canningensis*. However, results were not as clear at opposite extremes. *Kimberleyichthys* sp. had lower relative microstrain with the highest DNE of the sample, while *Bullerichthys fascidens* had higher stress during equivalent relative bite forces than expected, considering its DNE was closer to *Ca. concinnus* and *R. canningensis*. *Eastmanosteus calliaspis* and *T. pulchellus* had high values of both relative microstrain and DNE. *Incisoscutum sarahae* showed moderate values across all tests.

## Discussion

Our analyses revealed substantial interspecific variation in jaw stiffness and dental complexity among arthrodire placoderms from the Gogo Formation, demonstrating multiple mechanical pathways associated with hard-object feeding. Contrary to our first hypothesis (H1), the relatively narrow range of inferognathal mechanical advantage in our sample (~ 6%) did not closely correspond with variation in jaw performance or inferred durophagy across taxa. Mechanical performance of jaw structure was instead best distinguished by relative strain magnitudes (Fig. 2A), reflecting differences in resistance to bite-induced deformation. Our second hypothesis (H2) was also not supported. Dental surface complexity did not closely align with inferognathal strain magnitudes, because taxa with similarly low strain values exhibited both the lowest and highest complexity measures (Fig. 2B). In contrast, our third hypothesis (H3) received substantial support. Incorporating body size (Fig. 2A) clarified ecological distinctions among taxa and our results taken together suggest that predator-prey size relationships strongly influenced hard-object feeding adaptation in placoderms. These size relationships might also explain why taxa with similarly stiff jaws evolved markedly different levels of dental complexity.

Mechanical advantage does not appear to have played a strong role towards bite force adaptation in our Gogo Formation sample. There was certainly greater variation in mechanical advantage across all known placoderms than measured here, with a range more similar to what is seen in other stem gnathostomes^50^(see *Titanichthys* or *Alienacanthus*, for examples of particularly elongate inferognathals^51,52^. However, our sample demonstrates that mechanical advantage was not a necessary determinant of niche partitioning. Size-related (allometric) relationships^36,53^ appear to explain more of our results, particularly relating to the relative size of prey to predator.

Our findings indicate that placoderm durophagy encompassed at least two mechanically distinct feeding strategies: flat crushing surfaces were sufficient for crushing when prey could be engulfed and comminuted whole, whereas prey approaching or exceeding the functional oral cavity of the species tested required dental features capable of fragmentation, not crushing alone. While the smallest two species (*R. canningensis* and *Ca. concinnus*) both had stiffer jaws relative to size, and the flattest dental surfaces (see Fig. 1), comparisons of equivalent absolute bites indicated that they would still be limited to less resistant foods than larger species of the sample (Fig. 2A). With their lack of dentition inhibiting their ability to partition prey items into smaller pieces, the results indicate an ability to crush prey that were smaller than their oral cavity. However, *Kimberleyichthys* sp. had a different combination of characters that exemplified an ability to process shelled or armoured prey that could exceed its ingestive capacity.

*Kimberleyichthys* sp. had high jaw stiffness, and was also the largest species in the sample which greatly increased the absolute forces its jaw could withstand (Fig. 2A). Notably, it also had the highest dental surface complexity in the sample (Fig. 2B; Table 1). This elevated complexity was explained by a robust anterior odontode, alongside a tall rear dental arcade housing small teeth with rounded apices (see Fig. 1). Such an arrangement is reminiscent of the robust features of weapons designed to puncture and crush medieval armour, such as the war hammer or poleaxe (see images in^54,55^). These features together formed a dentition well suited to concentrating forces for initiating fracture of stiff materials. *Kimberleyichthys* also had a notably wide skull, and well-developed post ocular processes for bracing the neurocrania during feeding^56,57^. Altogether, these features suggest that prey crushing in *Kimberleyichthys* was not achieved through simple compression alone, but through armour or shell puncture and fragmentation of larger prey. Together, the contrasting patterns found between the smallest and largest species suggest that durophagy in Devonian arthrodire placoderms did not correspond to a single mechanical solution, but was rather multiple configurations related to predator-prey size relationships.

Our results for *Ca. concinnus* challenge some prior conclusions regarding this species. Previous linkage modelling and comparative two-dimensional finite element analyses of *Ca. concinnus* suggested a relatively weaker jaw than other placoderms, with elevated stress in the anterior inferognathal region. This has been interpreted as evidence for fine prey manipulation rather than durophagy^10,21^. However, when the three-dimensional morphology is considered^58^, the mediolaterally broad inferognathal blade and biting surface provided a more rounded (ellipsoidal) cross-sectional geometry, better suited to disperse bite-induced stresses. These results instead suggest a capacity for crushing structurally resistant prey. This is because resistance to bending scales with both dorsoventral height and mediolateral width (albeit to a lesser extent) through the equation for the second moment of area^59–61^. Similar to what is observed in mammalian skulls, broad, rounded jaw shapes in placoderms increased internal bone cross-sectional area available for force dispersion and better resisted torsional loading^14,19,36,62^. In terms of stress resolution, the placoderm inferognathal thus appears to have operated in a mechanically similar fashion to the canine teeth of carnivorans, where species producing higher bite forces tend to have canine teeth with mediolaterally broader dimensions^42,59^.

A more durophagous diet for *Ca. concinnus* is corroborated by other aspects of camuropiscid morphology indicative of durophagy, such as the fusion to the headshield of the reduced postsuborbital and submarginal plates which support the mandibular joint^63^, and the robustly ossified ethmoid capsule [AF1.1] with broad closely-packed, upper supragnathals^64,65^. *Rolfosteus canningensis*, the smallest taxon in our sample, shows a similar mechanical signature. However, its higher absolute strain under equivalent bite forces suggests it was likely restricted to a narrower range of smaller prey-sizes or prey with weaker structural defences. Its slightly elevated dental complexity relative to *Ca. concinnus* derived from a sharper anterior biting edge, which may have facilitated fine-scale prey selection or extraction from substrates^9^. Both *R. canningensis* and *Ca. concinnus* belong to the same family, the Camuropiscidae^66^, suggesting the possibility of some contingent factors behind their edentulate inferognathals which may have constrained dietary ecology in this group.

Several other species tested appeared better suited to slicing through softer tissues. *Co. croucheri*, *E. calliaspis*, and *T. pulchellus* combined higher relative strains with higher values of DNE, reflecting a relatively weaker jaw in association with sharper cutting edges. A slicing edge of the inferognathal and hard object feeding are not necessarily mutually exclusive in placoderms, provided there is sufficient body size, or that both inferognathal and dental arcade are sufficiently reinforced (see *Dunkleosteus terrelli*^21,67,68^. However, our results suggest a more generalist diet for the larger-bodied *E. calliaspis*^9^, better suited to slicing through the armour of smaller prey or the softer flesh of larger prey. The inferognathal of *Co. croucheri* only performed moderately during simulations of equivalent bite force, relative to size, and poorly under absolute bite force loading. Therefore, while its inferognathal was deeper with its larger second moment of area interpreted to support stronger bites^9,21^, our results suggest a selective diet limited to softer tissues, or smaller, less mobile prey. The high dental complexity with sharp slicing edges in *Co. croucheri*^30^ might indicate an ability to slice apart soft tissues in smaller active prey or when scavenging from carcasses of any size. Despite its medium size in our sample, *T. pulchellus* had the weakest inferognathal in the sample under both loading regimes. However, its high dental complexity highlighted the sharpness of its well-developed dentition, which likely allowed it to effectively slice through soft tissues. Our results do not support the ability of *T. pulchellus* to capture or break down particularly large or armoured prey items.

A finding of lower dental complexity and lower jaw stiffness in *B. fascidens* seems counterintuitive and may be related to contingency effects. The flat dental surface of the inferognathal and rounded supragnathals have been described as a unique mortar-and-pestle configuration well-suited for crushing^9^. Yet, its weaker jaw suggests a generalist diet or a diet limited to less resistant materials. Its crushing-like dentition may instead reflect retained ancestral morphology despite a shift toward more generalist feeding^36,69^.

While *I. sarahae* performed moderately in jaw stiffness and dental complexity, the DNE maps also clearly visualise fossa-like pits along its biting surface. These might be resorption pits where teeth were previously positioned (48), or wear facets potentially accumulated from grinding invertebrate prey or mixed with sediment over time. Such actions would potentially involve long-axis rotation of the inferognathal (46), and future microwear testing of its occlusal surface could confirm such activity.

Importantly, the anatomical and mechanical attributes described and discussed above are not an indication of specific diet composition, as this would have been strongly influenced by short-term shifts in productivity and species diversity. More robust features related to durophagous diets instead offer evidence of fundamental niche partitioning, as they indicate a capability to incorporate more resistant food resources that would be inaccessible to species with more fragile jaws. This is because morphology of the gnathostome feeding system tends to be largely predicted by the most mechanically challenging biting behaviours^14,20,70^, but these can be significantly influenced by opportunistic feeding or by an evolutionary history of accessing less desirable resources that are exploited seasonally or during periods of low productivity (i.e., fallback foods^71,72^. Despite this caveat, our findings demonstrate that adaptation to hard-object feeding in placoderms encompassed fundamentally different mechanical solutions depending on the relative size of prey to predator. Predator-prey size relationships have also played a dominant role in the evolution of talon shape in birds of prey (raptors)^73^, and crab claw morphology^74^, and the patterns discussed here will provide another dimension to the well-studied relationships between skull morphology and prey size^10,44,45,75,76^ in future research.

## Methods

Specimens were either Micro-CT scanned using the Flinders Tonsley Nikon XT H 225ST CT Scanner (Large-Volume Micro-CT System), with isotropic pixel sizes ranging from 50 to 65 μm, or surface scanned using an Artec Spider II handheld scanner (Artec 3D). Our sample included a *B. fascidens* (SAM P53771) inferognathal (IG) and suborbital (SO); *Ca. concinnus* (IG and SO: SAM P53772); *Co. croucheri* (IG: WAM 86.9.702, SO: SAM P53772); *E. calliaspis* (IG and SO: WAM 91.4.1); *I. sarahae* (IG and SO: WAM 01.11.14); *Kimberleyichthys* sp. nov. (IG and SO: WAM 2026.2.5); *R. canningensis* (IG and SO: WAM 74.2.56); *T. pulchellus* (IG: WAM 91.4.31, SO: *Torosteus* sp. indet. SAM P50606).

### Finite element analysis

We imported µCT data into Simpleware (Synopsis v. 2024.12), and generated 3D surface meshes of the IG and SO bones to model the position of the adductor mandibulae muscle origin and insertions.

The IG of one species, *Kimberleyichthys* sp., required reconstruction by merging the intact portion anterior to the cheek teeth of one IG with the intact rear blade and cheek teeth of the other, mirrored IG. This was done using Geomagic Wrap (3D Systems 2021.2.1.3026).

For each model, the IG was centred about geometric zero and then oriented to align the biting regions perpendicular to the z-axis in Blender software v. 4.5 (www.blender.org). This is also the orientation required for calculating Dirichlet normal energy (see below). The SO was then positioned for each model to produce an estimated gape angle of 20–25 degrees at the primary front tooth (odontode). This aligned the adductor muscle origin to record its centroid coordinates for applying muscle forces using Boneload (see below).

We exported the inferognathal meshes from Blender and converted to them to FEMs in Simpleware. Each model consisted of approximately 0.5 million 3D tetrahedral elements. Models were then imported into Strand7 (v.R3.1.3.a) for finite element modelling.

We assigned homogeneous, isotropic material properties previously used in finite element modelling of placoderm IGs (Young’s modulus: E ≈ 20 GPa; Poisson’s ratio: ν = 0.3)^21^. As is common practice in comparative FEA studies^19,22,77,78^, we emphasise that our results should be considered in a relative context and not as actual in vivo strain magnitudes experienced in life.

A total muscle force of 10 N was allocated to the adductor mandibulae of a randomly chosen specimen (*Kimberleyichthys* sp.). The absolute reference muscle force is arbitrary and does not change the relative differences of stress or strain between models^22,79^. This muscle force was scaled to IG volume of all other species to a 2/3 power^22,80^ to account for the allometric relationship between muscle force and size^81^(Table 2).

**Table 2:** Force scaling for placoderm inferognathal bite simulations. An initial muscle force (IF) was calculated for each species by scaling a value of 10 N for *Kimberleyichthys* to inferognathal volume by a 2/3rd power rule. Mechanical advantage (MA) was calculated by dividing the bite reaction force (BRF) by IF. For extrinsic bite forces in subsequent models, equivalent bite force relative to size (Hyp2) was calculated by multiplying IF by average MA, and equivalent absolute bite force was the average BRF of the initial simulations. Volume in mm^3^, Forces in Newtons.

Species	Volume	IF	BRF	MA	Hyp2	Hyp3
*B. fascidens*	1174.260	6.769	1.330	0.196	1.477	1.170
*Ca. concinnus*	408.196	3.346	0.712	0.213	0.730	1.170
*Co. croucheri*	210.470	2.152	0.440	0.204	0.469	1.170
*E. calliaspis*	1709.642	8.695	2.035	0.234	1.897	1.170
*I. sarahae*	1279.078	7.166	1.360	0.190	1.563	1.170
*Kimberleyichthys* sp.	2108.749	10.000	2.485	0.249	2.182	1.170
*R. canningensis*	172.507	1.884	0.392	0.208	0.411	1.170
*T. pulchellus*	250.799	2.418	0.608	0.251	0.528	1.170
**AVE.**			1.170	0.218		

**Fig. 3:**
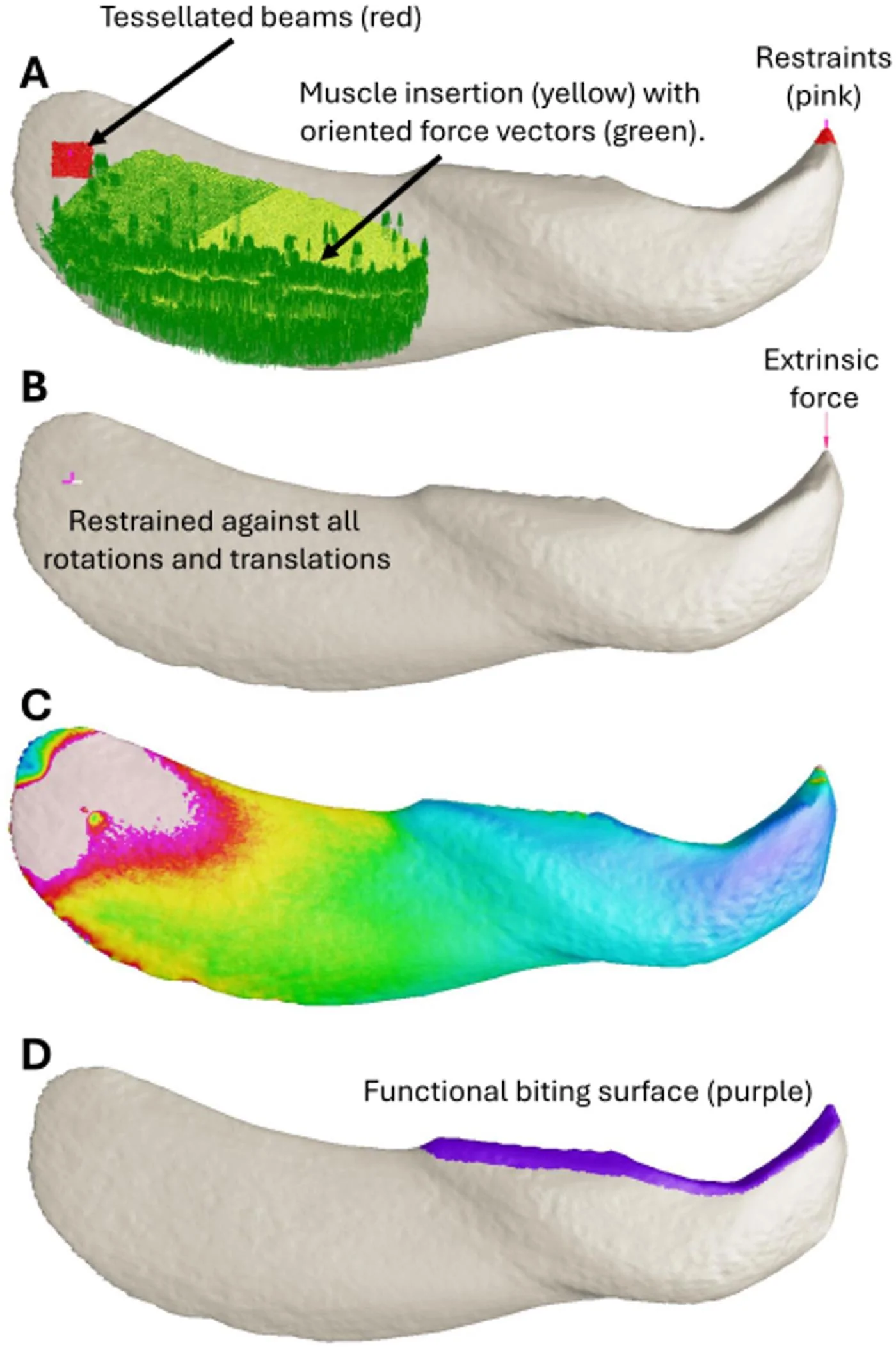
Modelling process shown on an inferognathal of *Bullerichthys fascidens*: (**A**) An initial simulation using an estimated muscle insertion was performed to calculate mechanical efficiency and bite reaction force. (**B**) Subsequent simulations used this initial data to predict equivalent bite force relative to size and equivalent absolute bite reaction force to apply as extrinsic forces on the front ‘tooth’ (**C**) Microstrain (*µε*), here set to a threshold of 200 *µε*, collected from each solved model. (**D**) The functional biting regions of each species (purple) were isolated to measure Dirichlet Normal Energy (DNE).

Muscle plates representing the adductor mandibulae origin and insertion were defined using Blender. While it is not entirely clear how this muscle inserted onto the IG or surrounding Meckel’s cartilage, it is generally understood that the musculature applied force to the lateral aspect of the IG blade between the point of rotation and the dental region^68^(Fig. 3A).

The masticatory muscle forces were applied to IG insertion plates using BoneLoad^82,83^. This software operates through MATLAB version 9.6 (R2019a) and orients the forces from the muscle insertion to the centroid of its origin. The loaded plates were imported into Strand7 and zipped to the nodes of their corresponding elements (Fig. 3A).

A single node on the tip of the primary anterior “tooth”, or odontode, was restrained against translation in the vertical axis. Rotation of the placoderm inferognathal occurred via an attached articular bone that was not modelled as it would be a separate element. However, the expected point of rotation in arthrodires inferognathals lies at the rear of the blade, as part of the surrounding articular joint^68^(Fig. 3A, B). Two nodes at each point of rotation, one on both sides of the IG, was restrained against translation for all axes. To reduce artifactual inflated instances of strain, the bone surrounding these restraints was then reinforced with tessellated beams given the with properties of structural steel (Fig. 3A). The models were then solved as linear static. From these models, the mechanical efficiency was estimated by dividing the bite reaction force by the input muscle force^22^.

Given that we only applied a single muscle, the adductor mandibulae, and that this muscle sits oblique at the dorsolateral aspect of the inferognathal, this led to unnatural lateral bending of the inferognathal. To better represent the dorsoventral motion of the IG, we instead used an approach more similar to previous FEA on placoderm jaws^21^, by applying the subsequently rescaled bite reaction forces (see below) to the front tooth as an extrinsic dorsoventral force on the biting tooth (Fig. 3B), while restraining the point of rotation against all translations and rotations. This better simulated a downward bite at the tooth, but also resulted in a slightly different focus, treating each inferognathal as a beam bending under an external load, rather than through modelled muscle activity. Since the study focus was on comparing bending under standardised loading, we considered this an acceptable and more accurate set of conditions.

We used the comparative FEA framework of Mitchell et al.^22^ to address two hypotheses: For a Type II hypothesis: comparing bite force adaptation, the average mechanical advantage of the sample was multiplied by the initial muscle input force of each model to generate an estimated bite reaction force of standardised mechanical advantage for each species (Table 2). This value was applied as an extrinsic vertical force to the odontode, and compared how each IG can support an equivalent bite force, relative to its size. For a Type III hypothesis: ecological biting ability, we applied the average sample bite reaction force from the initial simulations to all models as an extrinsic force (Table 2). This approach compares how well each IG can support an identical absolute bite force. While this latter method can be sensitive to intraspecific size variation as well^22^, the use of adult specimens was expected to mitigate these effects to a reasonable degree.

We used von Mises microstrain (µε) to examine performance (Fig. 3C). We extracted strain magnitudes from all elements of each model and removed the upper 1% of stress values, because these typically relate to artifactually inflated regions close to the restraints^22,84,85^. We plotted histograms of each simulation that depicted mean element von Mises microstrain for each model during both simulations.

### Dirichlet normal energy

We quantified dental arcade complexity using the MolaR R package (version 6.0)^49^. For each specimen, the functional dental arcade was first isolated from the remainder of the IG mesh (Fig. 3D). Unlike most applications of Dirichlet Normal Energy (DNE), which focus on the occlusal surfaces of individual mammalian molar teeth, our approach captures variation across the entire dental arcade. This allows complexity to be assessed from overall surface topography rather than from discrete tooth cusps or direct food-contact surfaces alone. In this context, low DNE values indicate relatively flat, simple crushing surfaces, whereas higher values reflect increased topographic complexity arising from combinations of teeth, ridges, and relief, not solely as a measure of sharp cutting edges as is typical in mammalian molars. Because DNE values are sensitive to mesh resolution, all dental surfaces were remeshed in Geomagic to a standardised resolution of 9,500–10,000 triangles prior to analysis. DNE was then calculated for each mesh using the ‘DNE’ function, and spatial patterns of surface complexity were visualised using the ‘DNE3d’ function, with heatmaps scaled to an identical maximum value of 1.

## Data Availability

STL files of all specimens will be made available on Morphosource ([https://www.morphosource.org/projects/000820205](https:/www.morphosource.org/projects/000820205)).
